# Editorial: Molecular perspectives on host-pathogen interactions in immune evasion, viral replication, and pathogenesis

**DOI:** 10.3389/fmolb.2026.1927973

**Published:** 2026-07-24

**Authors:** Nitin Sharma, Safder S. Ganaie, Deepak Kumar, Chandrabose Selvaraj

**Affiliations:** 1 Department of Pathology and Immunology, Washington University School of Medicine, St Louis, MO, United States; 2 Department of Molecular Genetics and Microbiology, College of Medicine, University of Florida, Gainesville, FL, United States; 3 Department of Medicine, Section of Infectious Diseases, Baylor College of Medicine, Houston, TX, United States; 4 CSRDD Lab, Bioinformatics Division, Department of Marine Biotechnology, AMET University (Deemed to Be University), Chennai, Tamil Nadu, India

**Keywords:** computational virology, host immune response, molecular pathogenesis, molecular virology, viral infection, viral replication

Pathogens have evolved sophisticated strategies to hijack host cellular machinery to establish productive infection and promote disease. Comprehensive understanding of host-pathogen interactions is central to infectious disease research as it reveals the molecular mechanisms by which pathogens invade host cells, manipulate cellular signaling networks, and evade the host immune response to drive infection. This understanding provides insight into the molecular dynamics involved in pathogenesis. Despite remarkable advances in biology, immunology, systems medicine, and structural biology, significant knowledge gaps remain in our comprehension of key pathogenic processes, including microbial entry, viral replication, immune evasion, and pathogen-induced tissue damage.

This Research Topic, “Molecular Perspectives on Host-Pathogen Interactions in Immune Evasion, Viral Replication, and Pathogenesis,” addresses these questions by bringing together studies that elucidate the emerging molecular mechanisms underlying infectious diseases, pathogen-driven pathologies, and innovative therapeutic strategies. The contributing articles span diverse disciplines, such as immunology, systems biology, nanotechnology, natural product pharmacology, transcriptomics, RNA biology, precision medicine, and artificial intelligence, reflecting the increasingly multidisciplinary nature of infectious disease research. This Research Topic primarily focuses on the molecular interplay between host-pathogen interactions and cellular systems to provide important insights into the integrated understanding and support the development of next-generation diagnostics, therapeutics, and preventive strategies.

Mecocci et al. employed integrated transcriptomic profiling to investigate equine sarcoids predominantly associated with bovine papillomavirus type 1 (BPV1). The authors identified broad alterations in gene and microRNA expression patterns, novel chimeric transcripts, and oncogenic signaling pathways that are potentially involved in viral infection-driven tumorigenesis. These findings offer valuable insight into host-pathogen interactions at the transcriptional level for a deeper understanding of virus-associated oncogenic mechanisms (Mecocci et al.). In a contributed review article, Radhamanalan illustrated the complexity of host-microbe interactions and explored the precision oral microbiome engineering via postbiotic and bacteriophage-based interventions that target *Streptococcus mutans* in dental caries. The integration of multi-omics approaches, systems biology, and microbiome ecology highlights the promising approaches aimed at restoring microbial homeostasis while retaining beneficial microbe populations. These precision-based applications demonstrate a vital transition from a broad spectrum of antimicrobial therapies to targeted, ecosystem-based interventions (Radhamanalan).

The study carried out by Garg et al. evaluated the combination of artificial intelligence and nanotechnology for next-generation viral diagnostics and pathogen surveillance. Integrating recent advances, such as machine learning algorithms, with nanotechnology approaches, including nanosensors and lab-on-chip technologies, provided favorable opportunities for rapid, scalable, and sensitive viral detection. These innovative findings have the potential to transform public health preparedness through the use of outbreak prediction, real-time monitoring, and personalized diagnostic solutions (Garg et al.). Wang et al. reported a comprehensive overview of the basic molecular regulation of and interaction between small RNAs and R-loops. They provided an overview of the molecular entities associated with the regulation of gene expression, genome stability, and DNA repair processes. Importantly, dysregulation of these pathways is involved in genomic instability, neurodegenerative disorders, and cancer progression. The evolving connection between disease pathogenesis and RNA-mediated regulation underscores the emerging importance of RNA biology in infectious diseases. The emergence of immune regulation is another key concept across this Research Topic (Wang et al.).

Zong et al. investigated the dual immune-modulatory regulation of microglia during viral encephalitis, which demonstrated their capacity to have both protective and detrimental antiviral effects on neuroinflammatory responses. The authors highlighted the significance of the heterogeneity of microglial activation and emphasized therapeutic modulation aimed at preserving neuroprotection while limiting tissue damage. Their review discussed the complexity of the immune response within the central nervous system during viral infection and infection-associated pathology (Zong et al.). Chandra et al. provided a detailed review of coagulopathy in viral hemorrhagic fevers, including Marburg, Lassa fever, Ebola, and Crimean-Congo hemorrhagic fevers. The authors explained how viral infections influence endothelial dysfunction, tissue factor activation, cytokine storms, and platelet abnormalities. In addition, they discussed disseminated intravascular coagulation and fibrinolysis dysregulation; importantly, they highlighted emerging therapeutic applications that target coagulation pathways, inflammatory mediators, and endothelial injury to investigate mechanisms involved in novel clinical interventions. The review clearly discusses pathogen-directed therapies and highlights the therapeutic potential of bioactive small molecules from natural sources (Chandra et al.). He et al. contributed a comprehensive review of catapol, a plant-derived iridoid glycoside derived from the plant *Rehmannia glutinosa*. The authors summarized the anti-inflammatory, antifibrotic, antioxidant, neuroprotective, and metabolic regulatory properties of this iridoid glycoside. In addition, they discussed the recent advances in drug delivery systems and clinical development, demonstrating how novel therapeutic innovations are integrated into traditional natural products in modern translational medicine (He et al.).

Collectively, the contributions to this Research Topic underscore the importance of integrating molecular biology, computational science, immunology, and translational research to better understand infectious diseases and their wide range of pathological consequences. Whether examining viral oncogenesis, immune dysregulation, genome stability, advanced diagnostics, or natural product therapeutics, each contribution deepens our understanding of host-pathogen interactions and disease mechanisms. Emerging technologies, such as artificial intelligence, precision medicine, single-cell biology, and multi-omics, will continue to advance the field and encourage interdisciplinary innovation. We hope this Research Topic will stimulate future research and inspire innovative strategies for understanding and treating infectious diseases.

